# An essential role of RNF187 in Notch1 mediated metastasis of hepatocellular carcinoma

**DOI:** 10.1186/s13046-019-1382-x

**Published:** 2019-09-02

**Authors:** Lei Zhang, Jiewei Chen, Juanjuan Yong, Liang Qiao, Leibo Xu, Chao Liu

**Affiliations:** 1 Guangdong Provincial Key Laboratory of Malignant Tumor Epigenetics and Gene Regulation and Department of Biliary-Pancreatic Surgery, Sun Yat-sen Memorial Hospital, Sun Yat-sen University, 107 Yan Jiang West Rd, Guangzhou, 510120 China; 2 Department of Pathology, Sun Yat-Sen University Cancer Center, State Key Laboratory of Oncology in South China, Collaborative Innovation Center for Cancer Medicine, Guangzhou, China; 3 Department of Pathology, Sun Yat-sen Memorial Hospital, Sun Yat-sen University, Guangzhou, China; 4 Storr Liver Centre, Westmead Institute for Medical Research, University of Sydney at Westmead Hospital, Westmead, NSW 2145 Australia

**Keywords:** Hepatocellular carcinoma, RNF187, Notch signaling, Metastasis

## Abstract

**Background:**

Aberrant activation of Notch signaling has been causally linked to the metastasis of hepatocellular carcinoma (HCC), however the underlying molecular mechanisms are still poorly understood. RING finger protein 187 (RNF187) was recently revealed to be a driver of several cancers, but its expression pattern and biological function in HCC are unknown.

**Methods:**

The expression levels of Notch1 and RNF187 were assessed in two independent cohorts of HCC tissues, and modulation of Notch1 in HCC cells was performed to explore the regulatory role of Notch1 in HCC metastasis. RNA-sequencing (RNA-seq), bioinformatics analysis, luciferase reporter analysis, and chromatin immunoprecipitation assay (ChIP) were used to clarify the relationship between Notch1 signaling and its potential target Ring finger protein 187 (RNF187). Gain- and loss-of-function studies were used to dissect the role of Notch1-RNF187 signaling in promoting HCC metastasis. The impact of Notch1-RNF187 activity in determining clinical prognosis for HCC patients was evaluated by multivariate Cox regression.

**Results:**

By RNA-seq, luciferase reporter analysis, and ChIP assay, RNF187 was confirmed to be a direct transcriptional target of Notch1, as Notch1 could activate RNF187 promoter whereas the pro-migratory and pro-invasive effects of Notch1 were significantly attenuated by RNF187 knockdown. Meanwhile, RNF187 silencing could attenuate the Notch1-dependent epithelial-mesenchymal transition (EMT). Moreover, overexpression of RNF187 counteracted the inhibitory effect of Notch1 knockdown on cancer progression. Importantly, HCC patients with high level of hepatic Notch1 expression had shorter disease-free survival (DFS) than those with low level of hepatic Notch1 expression. Furthermore, patients with high level of Notch1 and RNF187 co-expression showed the shortest DFS. The expression level of Notch1 and RNF187 was an independent prognostic factor for HCC.

**Conclusions:**

For the first time we identified that RNF187 is an essential factor for Notch1 to promote invasion and metastasis of HCC. Of highly clinical relevance, we found that activation of Notch1-RNF187 correlates with a worse prognosis of HCC patients. These findings provide a solid foundation for developing novel strategies to tackle HCC metastasis.

**Electronic supplementary material:**

The online version of this article (10.1186/s13046-019-1382-x) contains supplementary material, which is available to authorized users.

## Background

Hepatocellular carcinoma (HCC) is one of the most prevalent and life-threatening malignancies globally [1]. Metastasis significantly contributes to the high mortality of HCC patients after surgical curative therapy [2, 3]. HCC metastasis occurs both inside and outside the liver. Extrahepatic metastasis have been reported in 13.5–42% of HCC patients [4]. The median survival time of HCC patients with extrahepatic metastasis are only 4.9–7 months [4–6]. Thus, it is of great significance to further identify the potential mechanisms of HCC metastasis, which may contribute to the detection and intervention of HCC metastasis in early stage and formulate novel approaches to treat metastatic HCC. Notch family is evolutionarily conserved and plays fundamental roles in diverse biological processes, ranging from self-reorganization to the differentiation [7]. Altered Notch pathway not only contributes to multisystemic developmental defects, but also to the development and progression of many types of cancer [8–10]. Although abnormal expression of the Notch pathway has been demonstrated in HCC [11, 12], the molecular pathways governing metastatic HCC are not fully characterized and requires further investigation.

Ubiquitination is one of the most abundant posttranslational modifications and is involved in basic cellular processes [13]. Its dysfunction is associated with a series of diseases, particularly in tumorigenesis [14]. E3 ubiquitin ligases confer the substrate specificity and selectivity of the ubiquitin-conjugating system, and are of critical importance in this process [15]. RING finger protein 187 (RNF187), also known as RING domain AP-1 co-activator-1 (RACO-1), is a RING domain-containing ubiquitin E3 ligase [16, 17]. RNF187, a c-Jun co-activator to growth factor signal, is essential for AP-1 function in cell proliferation [17]. A recent study reported that RNF187 promotes HCC metastasis through inducing epithelial-mesenchymal transition (EMT) of HCC cells [18]. Increased expression of RNF187 has been correlated with poor survival in many cancers [18, 19]. However, the detailed molecular mechanism of how RNF187 promotes HCC metastasis has not been elucidated.

In the present study, using RNA-sequencing (RNA-seq), we found that Notch1 up-regulated RNF187 expression in HCC cells, and Notch1-mediated HCC metastasis was attenuated when RNF187 was knocked down, whereas overexpression of RNF187 counteracted the inhibition of cancer progression mediated by Notch1 knockdown, suggesting that RNF187 is essential for Notch1-mediated HCC metastasis. Moreover, we found that Notch1-RNF187 association correlates with the prognosis of HCC patients, which may provide a promising strategy for the treatment of Notch1-driven HCC metastasis.

## Materials and methods

### Tissue samples

Two cohorts of HCC patients were enrolled in our study. A total of 150 pairs of paraffin-embedded HCC specimens and corresponding adjacent non-tumor liver tissues (ANLTs) were collected as cohort I from 150 patients who have received curative surgical therapy at the Sun Yat-sen Memorial Hospital (Guangzhou, China) from 2013 to 2015. Another group of tissues samples from 67 patients with HCC treated at the Sun Yat-sen University Cancer Center were included in the cohort II and were used for microarrays chips. Ethics approval of this study was granted by the Ethics Committee of the Sun Yat-sen University. Disease-free survival (DFS) were calculated from the date of liver resection to the date when recurrence or metastasis was detected or the last follow-up visit.

### Animal studies

All animal experiments were approved by the Institutional Animal Care and Use Committee of Sun Yat-sen University (Guangzhou, China). Four-weeks-old male BALB/C nude mice (Beijing Vital River Laboratory Animal Technology Co., Ltd., China) were housed under a dedicated SPF facility on a 12 h light/dark cycle, and were cared for according to the Guide of the Care and Use of Laboratory Animals (National Institutes of Health publication nos. 80–23, revised 1996) and the institutional ethical guidelines for animal experiments.

For spontaneous metastasis assays, HCCLM6-luciferase cells (5 × 10^6^) with or without Notch1 knockdown were injected into the left hepatic lobe of nude mice. The in vivo tumor metastasis was detected by bioluminescence with the IVIS imagining system (Caliper Life Sciences, Hopkinton, MA, USA) after masking the signal from primary xenografts. In the lung colonization assays, mice were injected intravenously with HCCLM6-shNotch1 cells, PLC/PRF5-Notch1 and their control cells (2 × 10^6^). Mice were euthanized at the indicated time after xenograft implantation, and livers and/or lungs were fixed in formalin and embedded in paraffin using a routine method. Lung metastasis were validated on H&E stained sections and lung metastatic foci were counted.

### Statistical analysis

Statistical analyses were performed using SPSS (version 16.0) and Graphpad Prism (version 7.0). Quantitative data were compared using the Student’s t-test or one-way analysis of variance (ANOVA) for at least three groups [20]. Categorical data were analyzed using the *Chi*-square test. Disease-free survival (DFS) curves were determined by the Kaplan-Meier and log-rank test [21]. The cox proportional hazards regression model was used to verify the independent risk factors based on the variables selected in univariate and multivariate analysis. Gene ontology (GO) analysis was performed when gene sets were submitted to the DAVID website (http://david.abcc.ncifcrf.gov/home.jsp). A two-tailed *P* value of less than 0.05 was considered as statistically significant. More details of the materials and methods in this study are described in the Additional file 1.

## Results

### Notch1 significantly correlates with HCC metastasis

**Fig. 1:**
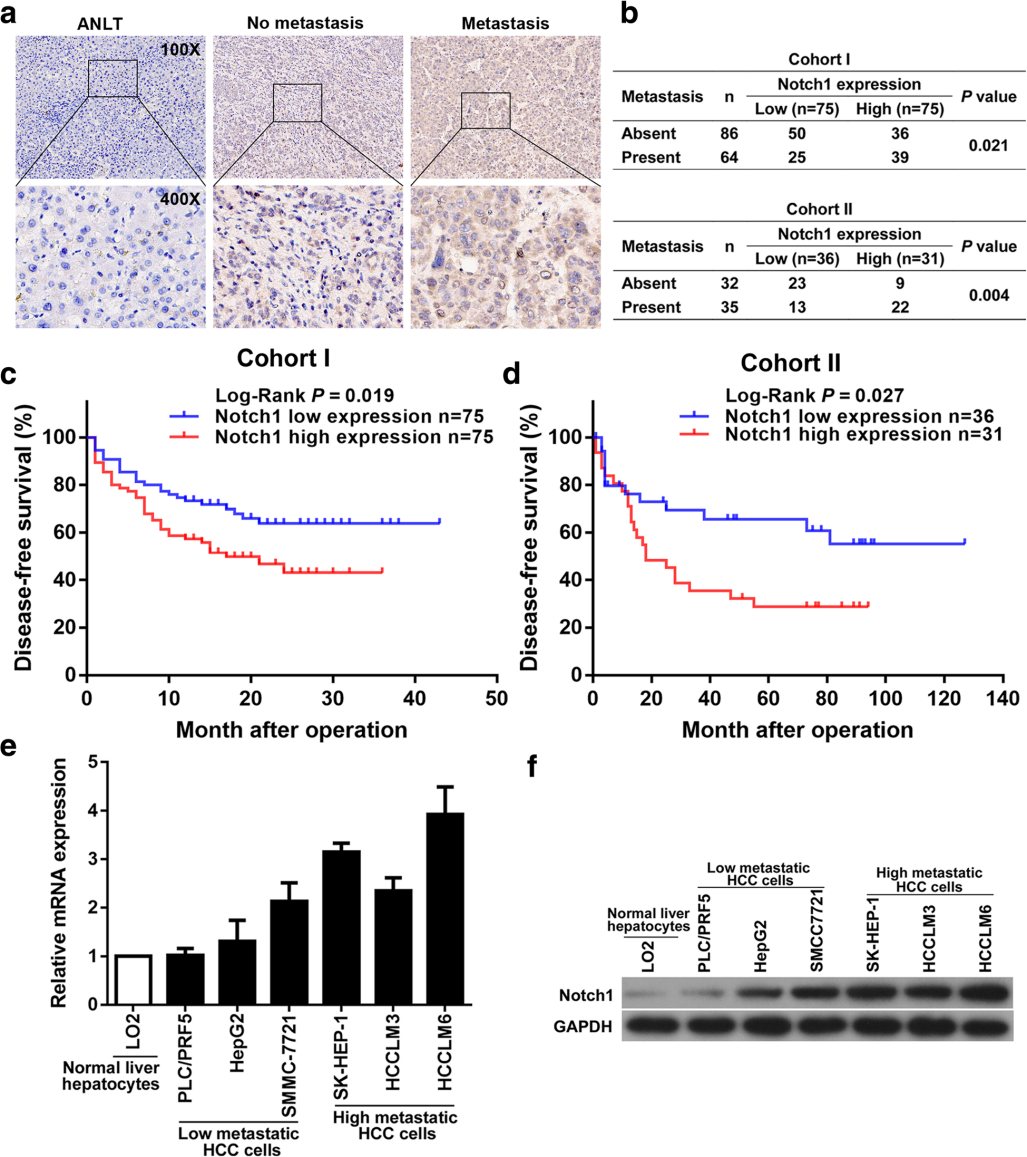
Notch1 correlates with HCC metastasis. **a** Notch1 expression in ANLT, no-metastasis and metastasis tumor tissue. **b** Correlation between Notch1 expression and metastasis of HCC patients in Cohort I and Cohort II was assessed by Pearson *chi*-square test. **c** DFS of HCC patients with high or low Notch1 expression in Cohort I and Cohort II (**d**). **e** Real-time PCR and (**f**) Western blotting assays of Notch1 expression in HCC cells

Immunohistochemistry (IHC) staining indicated that metastasis HCC tissues had the highest intensities of Notch1 staining, comparing with non-metastasis HCC tissues and ANLTs, (Fig. 1a). The correlation of Notch1 expression with HCC metastasis were estimated. It showed that high Notch1 expression was significantly correlated with metastasis in HCC patients from both cohort I and cohort II (Fig. 1b). Then, we analyzed the correlation of Notch1 expression with clinicopathological features of HCC patients. In the cohort I, it showed that high Notch1 expression was closely correlated with microvascular invasion in HCC patients, as shown in Table 1. Next, further survival analysis in cohort I showed that high Notch1 expression group had shorter DFS time than low Notch1 expression group (Fig.1c). Survival analysis for the cohort II also demonstrated this point (Fig. 1d).

**Table 1:** Association of Notch1 expression with clinicopathological factors of the HCC patients in cohort I and cohort II

Clinicopathological variables		Cohort I				Cohort II			
		n	Notch1 expression		^a^*P* value	n	Notch1 expression		^a^*P* value
			Low (*n* = 75)	High (*n* = 75)			Low (*n* = 36)	High (*n* = 31)	
Sex	Male	122	61	61	1.000	54	31	23	0.219
	Female	28	14	14		13	5	8	
Age, years	≤ 50	63	35	28	0.247	38	17	21	0.091
	> 50	87	40	47		29	19	10	
Serum AFP, ng/mL	≤ 20	63	33	30	0.620	23	12	11	0.853
	> 20	87	42	45		44	24	20	
Tumor nodule number	Solitary	123	60	63	0.524	52	28	24	0.972
	Multiple	27	15	12		15	8	7	
Maximal tumor size, cm	≤ 5	69	35	34	0.870	37	21	16	0.581
	> 5	81	40	41		30	15	15	
Microvascular invasion	Absent	68	40	28	**0.049**	39	24	15	0.130
	Present	82	35	47		28	12	16	
Tumor differentiation	Well	19	9	10	0.806	22	14	8	0.256
	Moderately or poorly	131	66	65		45	22	23	
TNM stage	I and II	97	49	48	0.864	47	26	21	0.689
	III and IV	53	26	27		20	10	10	

^a^ Pearson *chi*-square test was used for comparison between subgroups. Significant results (*P* < 0.05) are given in bold

Next, Real-time polymerase chain reaction (Real-time PCR) and Western blotting assays were performed to analyze the expression of Notch1 in HCC cells. Compared with the human normal liver LO2 cell, mRNA level of Notch1 was highly expressed in HCC cells (Fig. 1e). Increased protein expression level of Notch1 was further confirmed by Western blotting assays (Fig. 1f). Notably, the expression level of Notch1 in high-metastasis potential cell lines, such as SK-HEP-1, HCCLM3 and HCCLM6 was higher than that in low-metastasis potential cell lines PLC/PRF5, HepG2 and SMMC7721. Taken together, using multiple and complementary approaches in multiple samples including patient HCC tissues and HCC cell lines of low (PLC/PRF5, HepG2 and SMMC7721) and high (SK-HEP-1, HCCLM3 and HCCLM6) metastatic potentials, as well as using the survival analysis, we clearly demonstrated that increased expression of Notch1 correlates with increased metastasis of HCC.

### Notch1 promotes HCC cell invasion in vitro and metastasis in vivo

**Fig. 2:**
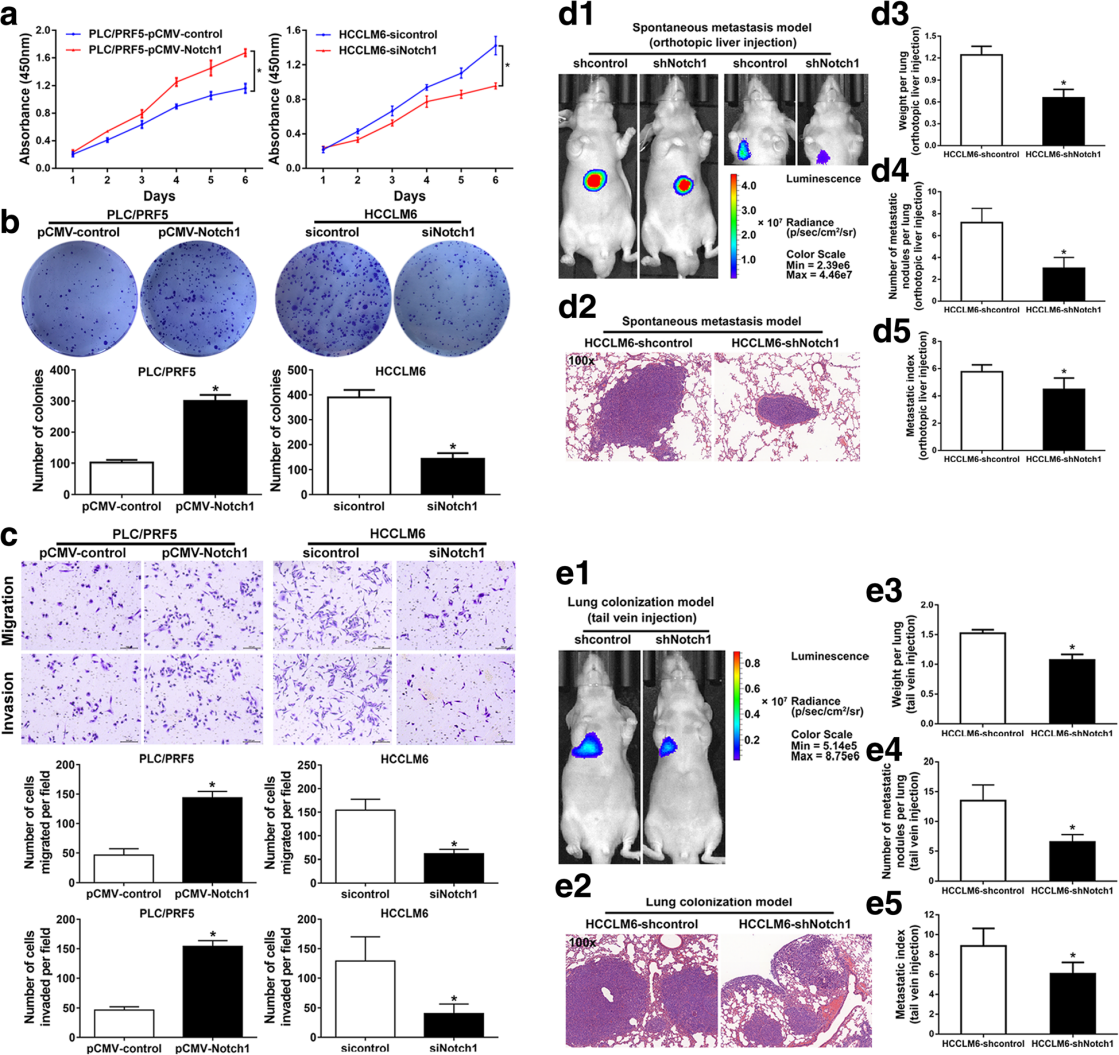
Notch1 promotes metastasis in HCC. Proliferation was examined by Cell Counting Kit-8 (CCK-8) (**a**) and colony formation assay (**b**). Migratory and invasive ability of Notch1-modulated cells were examined by Transwell assays (**c**). (**d**) In vivo spontaneous metastasis assay. HCC cells were injected into the left hepatic lobe of nude mice. (d1) Bioluminescence imaging of spontaneous metastasis was taken 10 weeks after orthotopic implantation. Right panel: bioluminescence imaging of metastasis taken after masking of the signal from primary xenografts. (d2) Lung metastasis was confirmed by H&E staining. (d3) Lung weights and (d4) number of lung metastatic nodules in each group. (d5) The metastasis index was calculated from the number of nodules/lung weight ratio. **e** In vivo lung colonization assays. The indicated stable cells were injected to nude mice via tail vein. (e1) Bioluminescence imaging and (e2) H&E staining of the lung metastatic tumors, respectively. (e3) Lung metastatic nodules, (e4) weights and (e5) metastasis index of nude mice in each group. *: *P* <  0.05

According to Notch1 expression level, we up-regulated Notch1 in low Notch1 expressing PLC/PRF5 cells by pCMV vector, named PLC/PRF5-pCMV-Notch1. High Notch1 expressing HCCLM6 cells were chosen to knock down Notch1 using small interfering RNA (siRNA), labeled HCCLM6-siRNA. The knockdown efficiency of three siRNAs for Notch1 was confirmed at both mRNA and protein levels through comparison with negative control (sicontrol) (Additional file 2: Figure S1). Among the three siRNAs tested, siRNA2 generated the most consistent knockdown results and was thus selected for further experiments. Next, we performed a variety of in vitro assays to evaluate the effect of Notch1 overexpression on HCC cell proliferation, migration and invasion. The results showed that Notch1 overexpression significantly promoted PLC/PRF5 proliferation by CCK-8 assays (Fig. 2a). Colony formation assays confirmed the effect of Notch1 overexpression on cell proliferation (Fig. 2b). Transwell assays showed that cell migration and invasion were significantly enhanced by Notch1 upregulation in PLC/PRF5 cells (Fig. 2c). Then we assessed the effect of Notch1 downregulation on HCCLM6 cells. The CCK-8 and colony formation assays showed that Notch1 knockdown significantly suppressed HCCLM6 cell proliferation (Fig. 2a and b). Results from transwell assays demonstrated that Notch1 downregulation impaired cell migration and invasion of HCCLM6 cells (Fig. 2c). These results indicated that Notch1 could be an influential role in the regulation of functions of HCC cell.

To study the role of Notch1 in metastasis of HCC, we manipulated Notch1 stable expression in HCC cells. Ectopic Notch1 was expressed in PLC/PRF5 cells, named as PLC/PRF5-Notch1. Meanwhile, short hairpin RNA (shRNA), sharing the same sequence with Notch1-siRNA2, was applied to knockdown Notch1 expression in HCCLM6 cells, named as HCCLM6-shNotch1. Expression level of Notch1 was confirmed by Real-time PCR and Western blotting assays (Additional file 3: Figure S2). Then, we established orthotopic xenograft tumor model and vein tail model, as previously described [22]. The HCCLM6-shNotch1 cells with stable knockdown of Notch1, as well as control cells, were implanted into nude mice to observe the metastasis of tumor cells in vivo. Orthotopic xenograft tumor model revealed that Notch1 knockdown inhibited tumor metastasis in vivo (Fig. 2d). Bioluminescence imaging showed that knockdown of Notch1 expression strongly inhibited the metastasis of HCCLM6 cells (Fig. 2 d1), which was further confirmed by H&E-stained sections (Fig. 2 d2). We further detected the metastatic nodules in lungs and lung weight (Fig. 2 d3 and d4). The lung metastasis rates in nude mice with tumors derived from HCCLM6-shNotch1 cells were significantly lower than that derived from control group (Fig. 2 d5). We also performed lung metastasis model by injecting cells into the lateral tail vein. As assessed by bioluminescence imaging (Fig. 2 e1) and H&E staining (Fig. 2 e2), Notch1 downregulation resulted in a decrease of numbers of lung metastatic nodules (Fig. 2 e3) and lung weight (Fig. 2 e4), as well as metastasis rates (Fig. 2 e5). The PLC/PRF5-Notch1 cells, as well as control cells, were implanted into nude mice to observe the metastasis of tumor cells in vivo through vein tail model. As assessed by H&E staining (Additional file 4: Figure S3a), Notch1 upregulation resulted in an increase of lung weight (Additional file 4: Figure S3b), numbers lung metastatic nodules (Additional file 4: Figure S3c), and metastasis rates (Additional file 4: Figure S3d). From the above findings, our studies showed that Notch1 could promote metastasis in HCC.

### Notch1 induces EMT in HCC cells

**Fig. 3:**
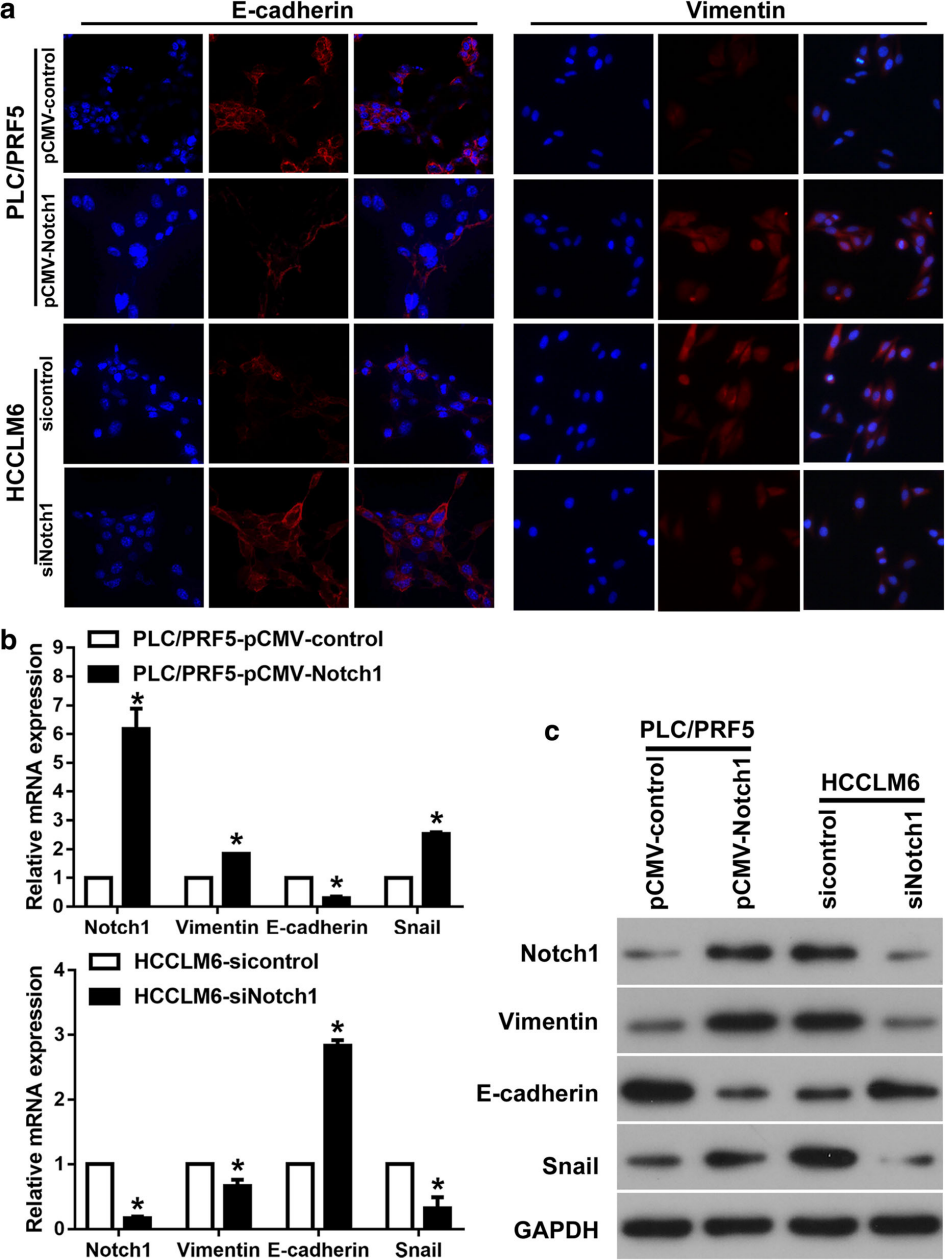
Notch1 induces EMT in HCC cells. **a** Immunofluorescent staining, (**b**) Real-time PCR, and (**c**) Western blotting assays of EMT markers in Notch1 modulated cells. *: *P* <  0.05

To investigate whether Notch1 promotes HCC invasion and migration by EMT, we evaluated epithelial and mesenchymal markers in HCC. Ectopic expression of Notch1 in PLC/PRF5 cells resulted in the decreased expression of E-cadherin and increased expression of Vimentin as evidenced by Immunofluorescence (IF); whereas Notch1 downregulation increased E-cadherin expression and decreased Vimentin expression in HCCLM6 cells (Fig. 3a). Consistent with the above findings, Notch1 upregulated cells showed decreased E-cadherin and increased Vimentin and Snail (EMT transcriptional factor) expressions at both mRNA and protein levels, whereas Notch1 downregulation induced the inverse results (Fig. 3b and c).

### RNF187 is a direct transcriptional target of Notch1

**Fig. 4:**
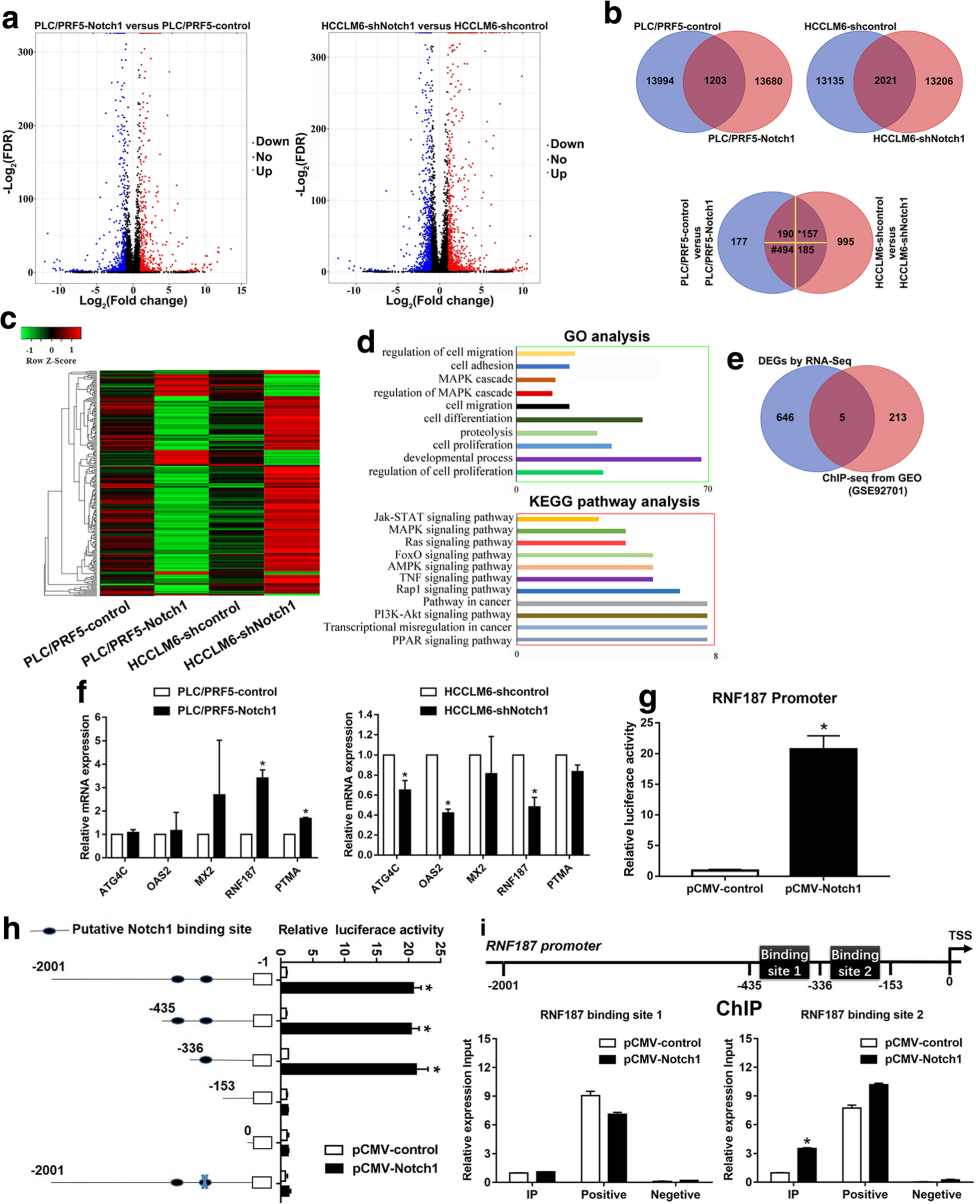
RNF187 is a downstream target of Notch1. **a** The scatter plot showed significant differentially expression genes (DEGs) of Notch1-modulated cells by RNA-seq; (**b**) Venn analysis was used to screen overlapping mRNA in step a; *: up-regulation in PLC/PRF5-Notch1 versus control group, simultaneously, down-regulation in HCCLM6-shNotch1 versus shcontrol group; #: down-regulation in PLC/PRF5-Notch1 versus control group, simultaneously, up-regulation in HCCLM6-shNotch1 versus shcontrol group. **c** Heat map and (**d**) GO categories, KEGG pathways enriched in the 651 DEGs from step b. **e** Venn diagrams to show intersections between RNA-seq gene sets (651 DEGs from step b) and ChIP-sequencing of Notch1 database (GEO accession no. GSE92701). **f** Real-time PCR was used to verify the DEGs. **g** Notch1 transactivates RNF187 promoter activity, as detected using a luciferase reporter assay. **h** Deletion and selective mutation analysis identified Notch responsive region in the RNF187 promoter, and relative luciferase activity was measured. **i** ChIP assay identified Notch1-binding sites in the RNF187 promoter. Real-time PCR was performed to detect the amounts of immunoprecipitated products. The positive control: anti-RNA polymerase II; the negative control: normal mouse IgG. *: *P* <  0.05

To further delineate the molecular basis by which Notch1 promotes metastasis in HCC, whole genome transcriptome analysis on PLC/PRF5-Notch1, HCCLM6-shNotch1, and their control cells were conducted using RNA-seq (Fig. 4a). Statistical analysis allowed the screening out of Differentially Expression Genes (DEGs) that exhibited highly significant differences according to the following criteria: DEGs increased in PLC/PRF5-Notch1 versus control group and simultaneously decreased in HCCLM6-shNotch1 versus shcontrol group; and vice versa, the expression of DEGs reduced in PLC/PRF5-Notch1 versus control group and simultaneously increased in HCCLM6-shNotch1 and shcontrol group. By defining a threshold of the cut-off as more than 1.5-fold change, 651 DEGs were screened (Fig. 4b. Additional file 5: Table S4), which mainly enriched regulation of cell migration, adhesion, etc. by Gene Ontology (GO) analysis, as shown in Fig. 4c-d. Kyoto encyclopedia of genes and genomes (KEGG) analysis showed that they activate pathways involved in PI3K-AKt pathway, transcriptional misregulation in cancer, etc. (Fig. 4d).

Next, we downloaded ChIP-sequencing database of Notch1 binding from GEO database (accession no. GSE92701) to further explore the underlying target genes of Notch1. Further Venn analyses with the above RNA-seq results showed that OAS2, ATG4C, RNF187, PTMA, and MX2 may potentially be direct transcriptional targets of Notch1 (Fig. 4e). Changes in these potential target genes were further confirmed by Real-time PCR (Fig. 4f). The results showed that Notch1 upregulation increased the expression of RNF187 and PTMA, and whereas Notch1 downregulation decreased ATG4C, OAS2, and RNF187 expression. Of particular significance was RNF187, which was upregulated 3.4-fold in response to Notch1 overexpression. Then we performed bioinformatic prediction of Notch1 transcription factor binding sites at promoter regions of RNF187 based on the ChIP-sequencing database (Additional file 6: Figure S4, highlight text, accession no. GSE92701). A luciferase reporter assay showed that Notch1 trans-activated RNF187 promoter activity (Fig. 4g). Sequence analysis revealed 2 putative Notch1 binding sites in the RNF187 promoter. Serial deletion and site-directed mutagenesis showed that the second Notch1 binding sites were critical for Notch1-induced RNF187 transactivation (Fig. 4h). A ChIP assay further confirmed that Notch1 binds directly to the RNF187 promoter (Fig. 4i). In our studies, we demonstrated that (1) Notch1 trans-activated RNF187 promoter activity; (2) among the two putative Notch1 binding sites in the RNF187 promoter, the second one was critical for Notch1-induced RNF187 transactivation; and (3) direct binding of Notch1 to the RNF187 promoter was demonstrated by ChIP assay. All these data clearly showed that RNF187 is a direct transcriptional target of Notch1.

### RNF187 is critical for Notch1-induced invasion and EMT in HCC

**Fig. 5:**
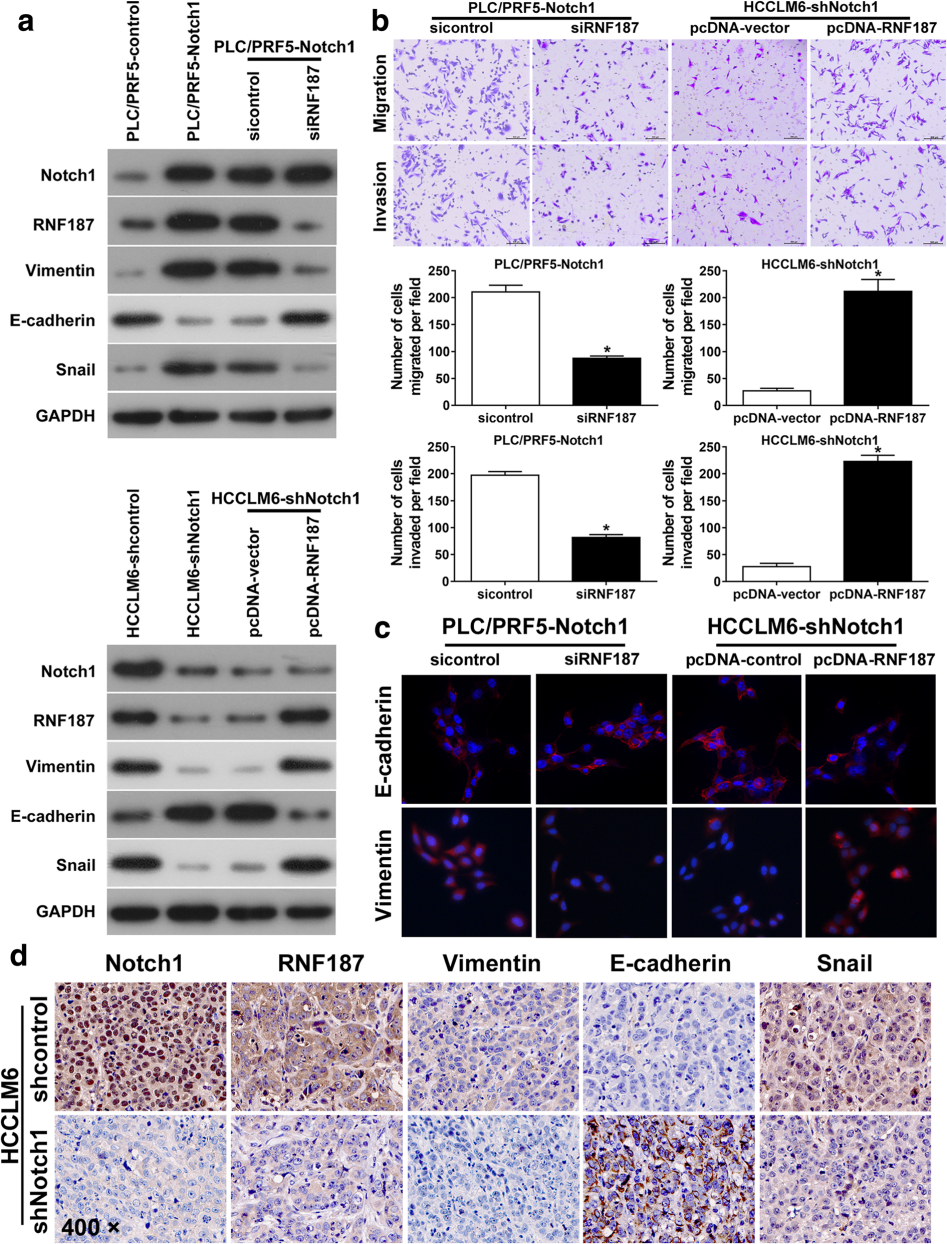
RNF187 is critical for Notch1-mediated HCC migration, invasion and EMT. **a** Western blotting assays were used to detect the expression of Notch1, RNF187, Vimentin, E-cadherin, and Snai1. **b** Migration and invasion assays in PLC/PRF5-Notch1 and HCCLM6-shNotch1 cells following RNF187 modulation. **c** Immunofluorescent staining assay of E-cadherin and Vimentin expressions in PLC/PRF5-Notch1 and HCCLM6-shNotch1 with RNF187 modulation. **d** Representative IHC images of Notch1, RNF187, Vimentin, E-cadherin, and Snail expression in the xenograft orthotopic liver tumors derived from HCCLM6-shNotch1 and their control cells. *: *P* <  0.05. Magnification: 400 ×

The overexpression of ubiquitin ligase E3C promoted HCC progression by regulating tumor cell EMT [23]. We assumed that RNF187 plays critical roles in Notch1 pathways effecting HCC cells. To test the assumption, first, we examined the level of RNF187 in Notch1-interfered HCC cells. Western blotting assays showed that ectopic expression of Notch1 increased RNF187 expression in PLC/PRF5 cells, whereas Notch1 knockdown inhibited RNF187 expression in HCCLM6 cells (Fig. 5a). To further test whether RNF187 was regulated by Notch1, RNF187-siRNA was transfected into PLC/PRF5-Notch1 cells, and RNF187 ectopic expression plasmid into HCCLM6-shNotch1 cells. The efficacy was confirmed by Real-time PCR and Western blotting assays (Additional file 7: Figure S5). Western blotting assays indicated that knockdown of RNF187 expression attenuated the loss of E-cadherin induced by Notch1 (Fig. 5a). In contrast, upregulation of RNF187 inhibited the increase in E-cadherin in HCCLM6-shNotch1 cells (Fig. 5a). Then, we measured the migratory and invasive capacity of Notch1-interfered HCC cells with RNF187 regulation. Transwell assays showed RNF187 knockdown decreased the migratory and invasive ability of Notch1 over-expression PLC/PRF5 cells (Fig. 5b). Next, IF assays showed that RNF187 knockdown increased E-cadherin and decreased Vimentin expression in PLC/PRF5-Notch1 (Fig. 5c). In contrast, upregulation of RNF187 in HCCLM6-shNotch1 cells had the opposite effect (Fig. 5c).

IHC analysis of liver orthotopic xenograft tumor of nude mice revealed that RNF187, Vimentin, and Snail expression levels were higher in tumors derived from HCCLM6-shcontrol cells than in tumors derived from HCCLM6-shNotch1. Whereas E-cadherin expression showed the inverse results (Fig. 5d). Taken the above results together, we concluded that RNF187 is critical for Notch1-mediated migration, invasion, EMT in HCC.

### Combination of Notch1 and RNF187 expression predicts HCC prognosis

**Fig. 6:**
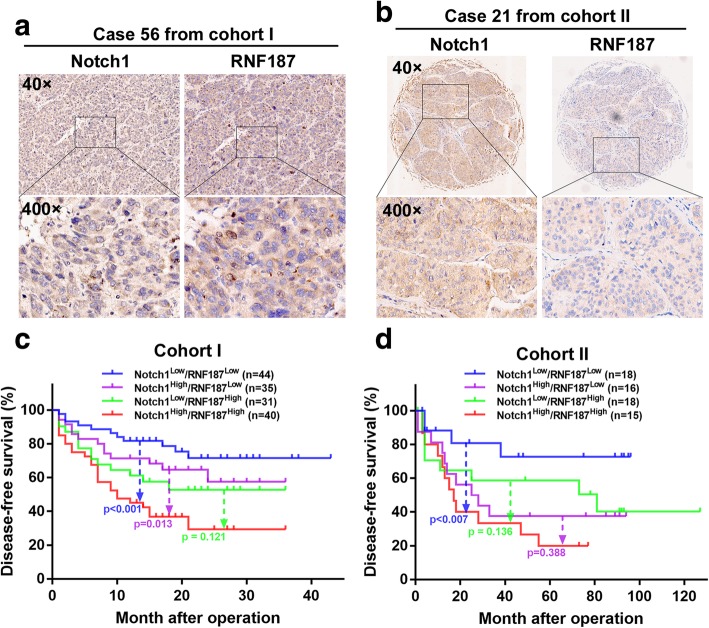
Patients with high expression of Notch1 and RNF187 had shortest DFS times. **a** Representative IHC images of Notch1 and RNF187 in consecutive sections from human HCC tissues in the cohort I and cohort II (**b**). **c** A Kaplan-Meier analysis of the correlation between the expression level of Notch1 and RNF187, and DFS time in the cohort I and cohort II (**d**)

We further evaluated the correlation between Notch1 and RNF187 in HCC tissues (Cohort I, Fig. 6a). Both the high expression of Notch1 and RNF187 were associated with aggressive tumor behavior (Tables 1 and 2). Patients were divided into four groups based on Notch1 and RNF187 expression levels. Kaplan-Meier analysis showed that patients with simultaneously high expression of Notch1 and RNF187 had the shortest DFS time (Fig. 6c). Multivariate analysis showed that the combination of Notch1 and RNF187 were risk factors for the DFS time (Table 3). The prognostic value of Notch1 and RNF187 were validated in an independent cohort II of 67 HCC patients by IHC staining (Fig. 6b, Tables 1 and 2). Similarly, the Notch1^High^/RNF187^High^ expression patterns had the shortest DFS time (Fig. 6d). Multivariate analysis also confirmed the above findings (Table 3).

**Table 2:** Association of RNF187 expression with clinicopathological factors of the HCC patients in Cohort I and Cohort II

Clinicopathological variables		Cohort I				Cohort II			
		n	RNF187 expression		^a^*P* value	n	RNF187 expression		^a^*P* value
			Low (*n* = 79)	High (*n* = 71)			Low (*n* = 34)	High (*n* = 33)	
Sex	Male	122	61	61	0.172	54	26	28	0.386
	Female	28	18	10		13	8	5	
Age, years	≤ 50	63	34	29	0.786	38	22	16	0.180
	> 50	87	45	42		29	12	17	
Serum AFP, ng/mL	≤ 20	63	35	28	0.546	23	6	17	**0.004**
	> 20	87	44	43		44	28	16	
Tumor nodule number	Solitary	123	66	57	0.604	52	26	26	0.820
	Multiple	27	13	14		15	8	7	
Maximal tumor size, cm	≤ 5	69	43	26	**0.029**	37	21	16	0.274
	> 5	81	36	45		30	13	17	
Microvascular invasion	Absent	68	40	28	0.169	39	19	20	0.695
	Present	82	39	43		28	15	13	
Tumor differentiation	Well	19	12	7	0.327	22	7	15	**0.030**
	Moderately or poorly	131	67	64		45	27	18	
TNM stage	I and II	97	59	38	**0.007**	47	21	26	0.128
	III and IV	53	20	33		20	13	7	

^a^ Pearson *chi*-square test was used for comparison between subgroups. Significant results (*P* < 0.05) are given in bold

**Table 3:** Univariate and multivariate analyses of risk factors associated DFS of HCC patients in cohort I and cohort II

Clinicopathological variables	Cohort I		Cohort II	
	HR (95% CI)	*P* value	HR (95% CI)	*P* value
Univariate analysis				
Age, years (> 50 vs. ≤50)	0.714 (0.437–1.166)	0.178	0.939 (0.483–1.827)	0.853
Sex (male vs. female)	1.268 (0.701–2.294)	0.433	0.696 (0.289–1.680)	0.421
Serum AFP, ng/mL (> 20 vs. ≤20)	1.785 (1.058–3.011)	**0.030**	0.689 (0.352–1.348)	0.277
Tumor nodule number (multiple vs. solitary)	2.912 (1.712–4.953)	**< 0.001**	1.247 (0.564–2.759)	0.585
Maximal tumor size, cm (> 5 vs. ≤ 5)	5.712 (2.978–10.955)	**< 0.001**	1.962 (1.006–3.829)	**0.048**
Microvascular invasion (present vs. absent)	6.060 (3.155–11.643)	**< 0.001**	1.223 (0.623–2.399)	0.558
Tumor differentiation (moderately or poorly vs. well)	12.369 (1.714–89.245)	**0.013**	0.879 (0.441–1.752)	0.714
TNM stage (III and IV vs. I and II)	4.739 (2.842–7.902)	**< 0.001**	0.865 (0.405–1.850)	0.709
Combination of Notch1 and RNF187				
A vs. D	0.276 (0.136–0.600)	**< 0.001**	0.230 (0.074–0.717)	**0.011**
B vs. D	0.451 (0.231–0.879)	**0.019**	0.680 (0.292–1.581)	0.370
C vs. D	0.599 (0.312–1.148)	0.123	0.557 (0.233–1.332)	0.188
Multivariate analysis				
Serum AFP, ng/mL (> 20 vs. ≤20)	1.164 (0.664–2.039)	0.596	NA	NA
Tumor nodule number (multiple vs. solitary)	1.818 (1.000–3.303)	0.050	NA	NA
Maximal tumor size, cm (> 5 vs. ≤ 5)	2.405 (1.085–5.333)	**0.031**	1.914 (0.937–3.913)	0.075
Vascular invasion (present vs. absent)	2.942 (1.413–6.125)	**0.004**	NA	NA
Tumor differentiation (moderately or poorly vs. well)	3.141 (0.395–24.985)	0.279	NA	NA
TNM stage (III and IV vs. I and II)	1.791 (0.928–3.457)	0.082	NA	NA
Combination of Notch1 and RNF187				
A vs. D	0.327 (0.156–0.685)	**0.003**	0.263 (0.084–0.828)	**0.022**
B vs. D	0.862 (0.425–1.748)	0.681	0.884 (0.363–2.154)	0.786
C vs. D	0.769 (0.375–1.576)	0.473	0.702 (0.283–1.738)	0.444

Significant results (*P* < 0.05) are given in bold. Combination of Notch1 and RNF187: A, Notch1 (low) & RNF187 (low); B, Notch1 (high) & RNF187 (low); C, Notch1 (low) & RNF187 (high); D, Notch1 (high) & RNF187 (high)

## Discussion

Tumor metastasis is a major contributor to HCC patient death. Notch family are critically important in various physiological processes and tumor progression, including liver cancer [11]. In clinical HCC tissues, upregulation of Notch1 has been observed, which is significantly associated with disease progression, such as HCC metastasis [24, 25]. Our current results provide accumulating evidence that Notch1 played an influential role in HCC metastasis. ChIP-sequencing analysis of Notch1 binding from GEO database indicated 218 genes that may involve in the Notch1 pathway to effect HCC. Among them, OAS2, ATG4C, RNF187, PTMA, and MX2 may potentially be direct transcriptional targets of Notch1. RNF187 is a gene upregulated significantly in response to Notch1 overexpression. Further investigation provided us the evidence that RNF187 was essential for Notch1 to promote HCC metastasis. Notch1-RNF187 association correlated with the prognosis of HCC patients, which might provide a promising strategy for the treatment of Notch1-driven HCC metastasis.

Aberrant Notch pathway activation contributes to multisystemic developmental defects and cancer development [8–10]. Notch1 promotes glioma cell migration and invasion through □-catenin and NF-κB pathway [26]. In prostate cancer, Notch1 silence inhibits invasion through matrix metalloproteinase-9 (MMP9) and urokinase plasminogen activator (uPA) [27]. Previous study indicates that Notch1 regulates metastasis of head and neck squamous cell carcinoma by inducing EMT [28]. Herein we reported that Notch1 expression was markedly higher in metastatic HCC tissues than non-metastasis tissues, and Notch1 expression was correlated with poor DFS, suggesting that Notch1 was a predictive factor for prognosis for HCC. The in vitro and in vivo experiments revealed that Notch1 could promote metastasis of HCC. EMT is a biological process that in which epithelial cells to obtain mesenchymal features, which results in reduced cell-cell contact, leading to increased motility and involving in facilitating metastasis of cancers [29–31]. Though controversial, EMT plays a crucial part in tumor metastasis including HCC [30, 32, 33]. Accumulated evidence implicates that Notch signaling has emerged as a key regulator for EMT [25]. Our study also showed that Notch1 ectopic expression had a significant effect on EMT, as indicated by the decreased epithelial marker expression and increased mesenchymal marker expression. In contrast, Notch1 downregulation showed opposite effects. These findings could be verified by expressions of Notch1, E-cadherin, and Vimentin in xenografted tumors.

Motivated by these results, we presented data regarding a novel Notch1 target gene involved with HCC metastasis: RNF187. In our studies, we demonstrated that Notch1 trans-activated RNF187 promoter activity; and direct binding of Notch1 to the RNF187 promoter was demonstrated by ChIP assay. All these data clearly showed that RNF187 is a direct transcriptional target of Notch1. The ubiquitin-proteasome family regulates a series of biological processes including signal transduction and proliferation [34]. The ubiquitination was found to be directly involved in human cancers progression including HCC [23, 35]. RNF187 is a RING domain-containing ubiquitin E3 ligase. An earlier study reported that in HCC, elevated RNF187 expression was associated with poor clinicopathological features and had shorter DFS, and overexpression of RNF187 resulted in enhanced EMT in HCC [18]. RNF187 is downregulated following nuclear factor kappa B (NF-κB) pathway inhibition in Late Erythroblasts [36]. Overexpression of RNF187 was shown to induce EMT and resistance to apoptosis in non-small-cell lung cancer cells through activation of MAPK and PI3K signaling [19]. These studies indicate that RNF187 is a key player in tumor progression. In this study, we observed that high expression levels of RNF187 was correlated with aggressive clinicopathologic features in HCC patients, including larger tumor size, tumor differentiation and TNM stage. Considering the critical roles of Notch1 and RNF187 expression in HCC tumorigenesis, we aimed to identify the underlying mechanisms responsible for the correlation between Notch1 and RNF187 expression in HCC cells. Luciferase reporter analysis, and ChIP assay confirmed that RNF187 was direct target of Notch1. Notch1-driven HCC progression could be reversed by depletion of RNF187, whereas overexpression of RNF187 counteracted the inhibition of cancer progression mediated by Notch1 knockdown.

In addition, the combination of Notch1 and RNF187 expression predicted prognosis for HCC, further validating the importance of the Notch1/RNF187 axis. The reason why many Notch1^High^/RNF187^Low^ patients were seen in our study was due to the statistical method used. In our study, Notch1 and RNF187 were divided into high- and low-expression groups using median cut-off values using the reported statistical methods [37]. As such, half of cases would have low- and the other half would have high expressions of Notch1 and RNF187, resulting in four different combinations in terms of the Notch1 and RNF187 expression levels. Data analysis may be affected by multiple factors, such as sample size, etiologies, ethnic backgrounds, and methods of statistical analysis. Cellular, molecular, and pathological heterogeneity are also important factors contributing to the study discrepancies. In our analysis, discrepancies did exist between the two cohorts in terms of clinicopathological data. However, Kaplan-Meier analysis clearly demonstrated that Notch1^High^/RNF187^High^ patients had the shortest DFS time (Fig. 6c-d), and this was supported by the multivariate analysis showing that the combination of Notch1 and RNF187 expression was a risk factor for patient survival (Table 3). Thus, both experimental and clinical evidence indicate that RNF187 is essential for Notch1 to promote HCC metastasis.

## Conclusions

In summary, our study clarified a key role of Notch1 and RNF187 in liver cancer metastasis. The results support that RNF187 inhibitors may offer an alternative therapeutic opportunity for HCC patients with high levels of Notch1.

## Additional files

Additional file 1: Supplementary materials and methods. (DOCX 80 kb)

Additional file 2: **Figure S1.** The efficacy of Notch1 silence and ectopic expression is determined in HCC cells. (**a**) Real-time PCR and (**b**) Western blotting assays confirmation of Notch1 mRNA and protein expression in PLC/PRF5-pcDNA-Notch1 cells, HCCLM6-siNotch1 cells and their control cells. *: *P* <  0.05. (TIF 85 kb)

Additional file 3: **Figure S2.** The efficacy of Notch1 silence or ectopic expression is determined in stably transfected HCC cells. (**a**) Real-time PCR and (**b**) Western blotting assays confirmation of Notch1 mRNA and protein expression in PLC/PRF5- Notch1 cells, HCCLM6-shNotch1 cells and their control cells. *: *P* <  0.05. (TIF 98 kb)

Additional file 4: **Figure S3.** In vivo lung colonization assays. The indicated stable transfectant cells were injected to nude mice via tail vein. (**a**) H&E staining of the lung metastatic tumors. (**b**) Lung weights, (**c**) metastatic nodules and (**d**) metastasis index of nude mice in each group. *: *P* <  0.05. (TIF 375 kb)

Additional file 5: **Table S4.** DEGs were screened. (XLSX 49 kb)

Additional file 6: **Figure S4.** Prediction of Notch1 binding sites at promoter regions of RNF187. Prediction of transcription factor binding sites at promoter regions of RNF187 based on the Chip-seq database of Notch1 binding from GEO (accession no. GSE92701). Yellow highlight text is binding site 1, and green is binding site 2. (TIF 482 kb)

Additional file 7: **Figure S5.** The efficacy of RNF187 silence or ectopic expression is determined in Notch1 mediated HCC cells. (**a**) Real-time PCR and (**b**) Western blotting assays confirmation of RNF187 mRNA and protein expression in RNF187 knockdown PLC/PRF5-Notch1 cells, RNF187 ectopic expression HCCLM6-shNotch1 cells and their control cells. *: *P* <  0.05. (TIF 97 kb)

## Data Availability

The data and materials of this study are available from the corresponding authors for reasonable requests.

## Abbreviations

AFP: Alpha-fetoprotein
ANLT: Adjacent non-tumor liver tissue
ChIP: Chromatin immunoprecipitation
DFS: Disease-free survival
EMT: Epithelial-mesenchymal transition
HCC: Hepatocellular carcinoma
IF: Immunofluorescence
IHC: Immunohistochemistry
shRNA: Short hairpin RNA
TNM: Tumor node metastasis
