# Supplementary material for: An essential role of RNF187 in Notch1 mediated metastasis of hepatocellular carcinoma
Source: J Exp Clin Cancer Res. 2019 Sep 2;38:384. doi: 10.1186/s13046-019-1382-x (PMC6720101; doi:10.1186/s13046-019-1382-x)
Supplement: Supplementary file 1 — Supplementary materials and methods. (DOCX 80 kb) [file 13046_2019_1382_MOESM1_ESM.docx]

**SUPPLEMENTARY MATERIALS AND METHODS**

**Cell lines and cell culture**

In this study, human HCC cell lines, PLC/PRF5, HepG2 and SK-Hep-1 cells were purchased from the American Type Culture Collection (ATCC, Rockville, MD, USA). Other human HCC cells SMMC7721, HCCLM3, and HCCLM6 were kindly provided by the Liver Cancer Institute of Fudan University (Shanghai, China). Immortalized human normal liver cells LO2 were purchased from the Cell Bank of Typical Culture Preservation Committee of Chinese Academy of Science (Shanghai, China). All cell lines were cultured in Dulbecco’s modified Eagle medium (DMEM, Gibco, Life Technologies, Carlsbad, CA, USA), supplemented with 10% fetal bovine serum (FBS, Gibco), 100 μg/mL penicillin, and 100 μg/mL streptomycin (Gibco), at 37°C in a humidified atmosphere with 5% CO_2_ incubator. PLC/PRF5, HepG2, and SMMC7721 are HCC cells with low metastatic potential, and HCCLM3, HCCLM6, and SK-Hep-1 have high metastatic potential [[1-5](#_ENREF_1)].

**RNA extraction and Real-time polymerase chain reaction (Real-time PCR).**

RNAiso Plus (Takara, Dalian, China) was used to extracted total RNA, and reverse transcription was performed using PrimeScript RT Reagent Kit (Takara), in accordance with the manufacturer’s protocol. The cDNA was subjected to Real-time PCR using the SYBR Green Kit (Takara) and the assay was performed on the Applied Biosystems 7900HT Fast Real-Time PCR System (Applied Biosystems, Foster City, CA, USA) according to the manufacturer’s recommendations. The cycle time (Ct) values of the selected genes were measured during the exponential amplification phase, and normalized with the value of GAPDH of the same sample. The relative levels of expression were quantified and analyzed using the 2^-ΔΔCt^ method. The expression level was normalized to the fold change that was detected in the corresponding control cells, which was defined as 1.0. All reactions were performed in duplicate. The primer sequences are listed in the Supplementary Table 1.

**Western blotting assay**

Total protein was extracted from cultured cells with ice-cold radioimmunoprecipitation assay (RIPA) lysis buffer (Beyotime, Nantong, China) with cocktail of proteinase and phosphatase inhibitors (Beyotime). The protein concentrations were quantified using the Bicinchoninic Acid Kit (Beyotime). An equal amount of protein samples was loaded in each lane, then separated by sodium dodecyl sulfate-polyacrylamide gel electrophoresis (SDS-PAGE), and transferred to polyvinylidene difluoride (PVDF) membranes (Millipore, Billarica, MA, USA). Nonspecific binding sites of the membrane were blocked with 5% nonfat milk in Tris-buffered saline-Tween (TBST) for 1 h at room temperature and incubated with the appropriate primary antibodies overnight at 4°C with gentle rocking. After washing with TBST, the membrane was incubated with the appropriate horseradish peroxidase (HRP)-conjugated secondary antibodies for 1 h at room temperature. After extensive washing with TBST, proteins were visualized by the enhanced chemiluminescence (ECL) detection kit in accordance with the manufacturer’s recommendations (Millipore). Antibodies used in this study are summarized in Supplementary Table 2.

**Construction of Tissue Microarrays and Immunohistochemistry (IHC) staining.**

Hematoxylin and eosin (H&E)-stained slides from all patients were reviewed and identified by two experienced pathologists, and the representative cores were premarked in the paraffin blocks. Tissue cylinders with a diameter of 1.0 mm were punched from the marked areas of each donor block and incorporated into a recipient paraffin block.

IHC staining for tissue was performed using the polymer HRP detection system (Zhongshan Golden bridge Biotechnology, Beijing, China) in accordance with the manufacturer’s instructions as described previously [[6](#_ENREF_6)]. The paraffin sections were dewaxed and antigen retrieval with 0.01 M sodium citrate buffer (pH 6.0), followed with 3 % hydrogen peroxide incubated for 10 min at room temperature and next goat serum blocking for 10min to block endogenous peroxidase. Sections were incubated with the primary antibody overnight at 4 °C in a humidified chamber, followed by HRP conjugated secondary antibody incubation for 30 minutes at room temperature. Antibody binding was detected by DAB and reaction was stopped by immersion of tissue sections in distilled water once brown color appeared. Tissue sections were counterstained by hematoxylin, dehydrated in graded ethanols and mounted. The antibodies were listed in the Supplementary Table 2.

The results of IHC staining were analyzed by two independent pathologists who were blinded to patients’ clinical outcome [[7-9](#_ENREF_7)]. The intensity of staining was scored as 0 (negative), 1 (weak), 2 (medium), and 3 (strong). The extent of IHC staining was based on the percentage of positive cells of positive tumor cells in the whole tissue slice. Intensity score and positive rate score were then multiplied to calculate the overall score. The protein expression was divided into high and low expression group by using median value as the cut-off for further analysis [[10](#_ENREF_10)].

**Plasmid,** **small interfering RNA and transfection**

pCMV-Notch1 was purchased from the Obio Technology (Shanghai, China) Corp., Ltd. The RNF187 cDNA was inserted into the pcDNA3.1 vector, which were purchased from Genechem (Shanghai, China). The small interfering RNAs (siRNAs) and their control (GenePharma, Shanghai, China) were used in this study. The target sequences are presented in Supplementary Table 3. Cells were plated at a density of 5 × 10^5^ cells/well in six-well plates and transfection was conducted at 70–80% confluence after 12-24 hours. Transfection used Lipofectamine 3000 (Invitrogen, Carlsbad, CA, USA) according to the manufacturer’s instructions. After 6 hours of transfection, cells were washed and allowed to recover overnight in fresh medium. At 48 h post-transfection, cells were harvested for assay.

**Cell Counting Kit-8 (CCK-8) and colony formation assay**

To determine the level of cell proliferation in vitro, CCK-8 (Dojindo Laboratories, Kumamoto, Japan) was used according to the manufacturer's instructions. Briefly, cells were seeded into each well of 96 well plates at a density of 5 × 10^3^ cells per well in a final volume of 100 μL medium. After culturing for 12 h, 100 μL fresh complete medium containing 10 μL CCK-8 solution was added into each well at different time points (1, 2, 3, 4, 5 and 6 days), and the absorbance at 450 nm wavelength was measured using a microplate reader (BioTek, USA) after incubation at 37°C for 2h to calculate the number of viable cells. For colony formation assays, cells were plated into a six-well cell culture plate at a density of 5 × 10^2^ cells per well and cultured for 2 weeks. The numbers of colonies per well were counted to evaluate cell proliferation after fixing in 4% paraformaldehyde, staining with 1% crystal violet. All assays were conducted at least three times independently.

***In vitro* migration and invasion assay**

A 24-well transwell plate (8 μm pore size, Corning, NY, USA) was used to measure the migratory and invasive ability of cells. For migration assays, 5×10^4^ cells in 200 μL of DMEM containing 0.1% FBS were placed into the upper chamber, and 600 μL medium containing 10% FBS was added to the lower chambers. For invasion assays, chamber inserts were pre-coated with 50 μL 1:8 mixture of BD Matrigel (BD Biosciences, San Jose, CA, USA) and DMEM for overnight under sterile conditions. Then 1×10^5^ cells were seeded in the upper chamber. After 24 h (migration assays) or 48 h (invasion assays), cells on the top side of each insert were scraped off gently, and then fixed in 4% paraformaldehyde, and stained by 0.1% crystal violet. Three random microscopic fields were counted per field for each group under light microscope. All assays were conducted at least three times independently.

**Establishment of Notch1** **stable transfectant cells**

Notch1 overexpression and knockdown lentivirus as well as their negative control lentivirus were purchased from GenePharma (Shanghai, China). Among the three siRNAs tested, siRNA2 presented the most consistent knockdown efficiency, which was used for the shNotch1 lentivirus package. Notch1 intracellular domain (NICD), activated form of Notch1 lentivirus was transfected into PLC/PRF5 cells, and lentiviral containing short hairpin RNAs (shRNA) targeting Notch1 was transfected into HCCLM6 cells. Lentivirus was infected into HCC cells with a multiplicity of infection (MOI) ranging from 30 to 50 in the presence of 5 μg/mL polybrene (Sigma-Aldrich, USA) according to the manufacturer’s instructions. Cells transfected with empty vector were used as controls. On day three after transduction, medium with 2 μg/mL puromycin (Sigma-Aldrich) was used to select stable cells. Pooled populations of stable transfectant cells, which were obtained two weeks after drug selection, were used in subsequent experiments. The target sequences of RNAi and cDNA clone are listed in the Supplementary Table 3.

**Immunofluorescence**

Cells grown on the culture slides (BD Biosciences) were maintained in a 24 well plates. After incubation for 24 h, the cells were washed with PBS and fixed with 4% phosphate-buffered neutral formalin in PBS for 10 min, permeabilized with 0.1% Triton X-100 for 10 min at room temperature, and then blocked with 1% bovine serum albumin (BSA) for 1 hour at room temperature. The cells were incubated with the indicated primary antibodies overnight at 4°C. After washing with PBS three times, cells were then incubated with appropriate secondary antibodies at room temperature for 1 h. The antibodies were listed in the Supplementary Table 2. Nuclei was stained with 4,6-diamidino-2-phenylindole DAPI (Beyotime) for 5 min. Following a final rinse of three times with PBS, images were captured using a Leica fluorescence microscope (Rueil-Malmaison).

**RNA-Sequence (RNA-Seq) and bio-informatic analysis**

Total RNA was extracted from HCCLM6 cells infected with lentiviruses expressing Notch1-shRNA and PLC/PRF5 cells infected with lentiviruses expressing Notch1 and their corresponding control cells using RNAiso Plus (Takara) according to the manufacturer’s protocol. Then, RNA quantity and quality were determined using a NanoDrop 2000 and an Agilent Bioanalyzer 2100 (Agilent, Germany). cDNA synthesis and library construction were carried out according to the manufacturer’s instructions, and sequenced using Illumina HiSeqTM 2500 by Gene Denovo Biotechnology Co. (Guangzhou, China). RNA-Seq reads were aligned to the human reference sequence National Center for Biotechnology Information (NCBI) hg38 with TopHat2 to calculate the values of fragments per kilobase of transcript per million mapped reads for known transcripts (FPKM). A two-class comparison of two group cell was performed to determine differentially expressed genes. We selected a fold change of 1.5 as the threshold for up-regulation or down-regulation, with a P-value < 0.05 of the fold-change being statistically significant. Statistical analysis allowed the screening out of Differentially Expression Genes (DEGs) that exhibited highly significant differences according to the following criteria: DEGs increased in PLC/PRF5-Notch1 versus control group and simultaneously decreased in HCCLM6-shNotch1 versus shcontrol group, and vice versa, the expression of DEGs reduced in PLC/PRF5-Notch1 versus control group and simultaneously increased in HCCLM6-shNotch1 and shcontrol group. To further explore the underlying target genes of Notch1, we downloaded Chip-seq database of Notch1 from GEO (accession no. GSE92701), and analyzed previously published gene expression profile downstream of Notch1.

**Luciferase report assay**

We analyzed the Notch1 binding regions of RNF187 based on the Chip-seq GEO dataset (Additional file 4: Figure S3, accession no. GSE92701). The RNF187 promoter construct (-2001/-1) RNF187 was generated from human genomic DNA corresponding to the sequence from -2001 to -1 (relative to the transcriptional start site) and cloned to the pGL3-Basic vector (Promega, Madison, WI, USA). The deletion constructs of the Notch1 promoter ([-435/-1] RNF187, [-336/-1] RNF187, and [-153/-1] RNF187) were mutated with a QuikChange II site-directed mutagenesis kit (Stratagene, La Jolla, CA). Cells were plated in 24-well plates and were co-transfected with 40 ng of reporter construct, 500 ng of expression vector, and 5 ng of the internal control Renilla construct (Promega) using Lipofectamine 3000 (Invitrogen). Luciferase activity was detected using the Dual Luciferase Assay (Promega) according to the manufacturer’s instructions after 36h.

**Chromatin Immunoprecipitation (ChIP) assay.**

ChIP assays were performed using the EpiQuik™ Chromatin Immunoprecipitation Kit (P-2002, Epigentek, NY, USA) according to the manufacturer’s protocol. Briefly, cells that were transfected with the appropriate plasmids, were collected and fixed for 10 min at 37 °C with 1% formaldehyde, followed in sequence with cell lysis buffer (50 mM Tris (pH 8.1), 10 mM EDTA, 1% SDS, and 1 mM PMSF) and DNA shearing, protein and DNA immunoprecipitation, cross-linked DNA reversal and DNA purification, and finally the immunoprecipitated DNA fragments were detected by Real-time PCR using appropriate primers (Supplementary Table 1). Immunoprecipitations included the positive control (Anti-RNA Polymerase II), and the negative control, (Normal Mouse IgG).

**SUPPLEMENTARY REFERENCES**

1. Bertran E, Crosas-Molist E, Sancho P, Caja L, Lopez-Luque J, Navarro E, et al. Overactivation of the TGF-beta pathway confers a mesenchymal-like phenotype and CXCR4-dependent migratory properties to liver tumor cells. Hepatology. 2013;58(6):2032-44.

2. Zhou YM, Cao L, Li B, Zhang RX, Sui CJ, Yin ZF, et al. Clinicopathological significance of ZEB1 protein in patients with hepatocellular carcinoma. Ann Surg Oncol. 2012;19(5):1700-6.

3. Chung KY, Cheng IK, Ching AK, Chu JH, Lai PB, Wong N. Block of proliferation 1 (BOP1) plays an oncogenic role in hepatocellular carcinoma by promoting epithelial-to-mesenchymal transition. Hepatology. 2011;54(1):307-18.

4. Tang ZY, Ye SL, Liu YK, Qin LX, Sun HC, Ye QH, et al. A decade's studies on metastasis of hepatocellular carcinoma. J Cancer Res Clin Oncol. 2004;130(4):187-96.

5. Tian J, Tang ZY, Ye SL, Liu YK, Lin ZY, Chen J, et al. New human hepatocellular carcinoma (HCC) cell line with highly metastatic potential (MHCC97) and its expressions of the factors associated with metastasis. Br J Cancer. 1999;81(5):814-21.

6. Wu F, Yang LY, Li YF, Ou DP, Chen DP, Fan C. Novel role for epidermal growth factor-like domain 7 in metastasis of human hepatocellular carcinoma. Hepatology. 2009;50(6):1839-50.

7. Yong KJ, Gao C, Lim JS, Yan B, Yang H, Dimitrov T, et al. Oncofetal gene SALL4 in aggressive hepatocellular carcinoma. N Engl J Med. 2013;368(24):2266-76.

8. Chu D, Li Y, Wang W, Zhao Q, Li J, Lu Y, et al. High level of Notch1 protein is associated with poor overall survival in colorectal cancer. Ann Surg Oncol. 2010;17(5):1337-42.

9. Zhou L, Zhang N, Song W, You N, Li Q, Sun W, et al. The significance of Notch1 compared with Notch3 in high metastasis and poor overall survival in hepatocellular carcinoma. PLoS One. 2013;8(2):e57382.

10. Han D, Li J, Wang H, Su X, Hou J, Gu Y, et al. Circular RNA circMTO1 acts as the sponge of microRNA-9 to suppress hepatocellular carcinoma progression. Hepatology. 2017;66(4):1151-1164.

**Supplementary Table 1** The primer sequences used in this study

| Primer name | Primer sequence |
| --- | --- |
| Primers for real-time PCR |  |
| Notch1 | F: GGTGAACTGCTCTGAGGAGATC |
|  | R: GGATTGCAGTCGTCCACGTTGA |
| GAPDH | F: GTCTCCTCTGACTTCAACAGCG |
|  | R: ACCACCCTGTTGCTGTAGCCAA |
| E-cadherin | F: GCCTCCTGAAAAGAGAGTGGAAG |
|  | R: TGGCAGTGTCTCTCCAAATCCG |
| Vimentin | F: AGGCAAAGCAGGAGTCCACTGA |
|  | R: ATCTGGCGTTCCAGGGACTCAT |
| Snail | F: TGCCCTCAAGATGCACATCCGA |
|  | R: GGGACAGGAGAAGGGCTTCTC |
| ATGC4 | F: TAGAGGATCACGTAATTGCAGGA |
|  | R: GTTGTCAAAGCTGAGCCTTCTAT |
| OAS2 | F: CTCAGAAGCTGGGTTGGTTTAT |
|  | R: ACCATCTCGTCGATCAGTGTC |
| MX2 | F: CAGAGGCAGCGGAATCGTAA |
|  | R: TGAAGCTCTAGCTCGGTGTTC |
| RNF187 | F: GTGATGGACCGTAGGAAGAAGG |
|  | R: GTGACCTGAACCGCTCAGTG |
| PTMA | F: GGAGGCTGACAATGAGGTAGA |
|  | R: TGGTATCGACATCGTCATCCT |
| Primers used for ChIP in the RNF187 promoter: |  |
| RNF187 binding site 1 | F: ATGAGAGCAGCGGAGGAGG |
|  | R: TGGGCTGTCCCTTCCTACTTC |
| RNF187 binding site 2 | F: GGGACAGCCCAGTAAGATTTCA |
|  | R: TGCGCTCCACAGACAAGATC |

**Supplementary Table 2** Antibodies used in this study

| Antigens | Manufacturers | Applications |
| --- | --- | --- |
| Notch1 | #3608, Cell Signaling Technology, Beverly, MA, USA | 1:1000 for WB |
|  | ab8925, Abcam, Cambridge, MA, USA | 1:200 for IHC |
| E-Cadherin | #3195, Cell Signaling Technology, Beverly, MA, USA | 1:1000 for WB  1:200 for IF  1:400 for IHC |
| Vimentin | AF7013, Affinity Biosciences, Cincinnati, OH, USA | 1:1000 for WB  1:100 for IF  1:200 for IHC |
| Snail | AF6032, Affinity Biosciences, Cincinnati, OH, USA | 1:1000 for WB |
|  |  | 1:200 for IHC |
| RNF187 | ab105034, Abcam, Cambridge, MA, USA | 1:1000 for WB |
|  | HPA030098, Sigma-Aldrich, St. Louis, MO, USA | 1:200 for IHC |
| GAPDH | #5174, Cell Signaling Technology, Beverly, MA, USA | 1:1000 for WB |
| HRP-linked anti-rabbit IgG | #7074, Cell Signaling Technology, Beverly, MA, USA | 1:5000 for WB |
| HRP-linked anti-mouse IgG | #7076, Cell Signaling Technology, Beverly, MA, USA | 1:5000 for WB |
| Alexa Fluor 594 goat anti-rabbit IgG | R37117, Life Technologies, Carlsbad, CA, USA | 1:200 for IF |

Abbreviations: WB western blotting, IHC immnuohistochemistry, IF immnuoflurorescence, HRP horseradish peroxidase.

**Supplementary Table 3** The sequences of RNAi and cDNA clone used in this study

| Name | Sequence |
| --- | --- |
| Notch1-siRNA1 | TGGCGGGAAGTGTGAAGCG |
| Notch1-siRNA2 | GGTGTCTTCCAGATCCTGA |
| Notch1-siRNA3 | GGACCAACTGTGACATCAA |
| RNF187-siRNA1 | GGUGGUCAGAUAUUAUGGUTT |
| RNF187-siRNA2 | GAGUGACAGAUUAAUGUAUTT |
| RNF187-siRNA3 | CCACCGUUCUACUCUCUAATT |
| Nucleotide Sequence of Notch1 (NICD) | CCTGAGGGCTTCAAAGTGTCTGAGGCCAGCAAGAAGAAGCGGCGGGAGCCCCTCGGCGAGGACTCCGTGGGCCTCAAGCCCCTGAAGAACGCTTCAGACGGTGCCCTCATGGACGACAACCAGAATGAGTGGGGGGACGAGGACCTGGAGACCAAGAAGTTCCGGTTCGAGGAGCCCGTGGTTCTGCCTGACCTGGACGACCAGACAGACCACCGGCAGTGGACTCAGCAGCACCTGGATGCCGCTGACCTGCGCATGTCTGCCATGGCCCCCACACCGCCCCAGGGTGAGGTTGACGCCGACTGCATGGACGTCAATGTCCGCGGGCCTGATGGCTTCACCCCGCTCATGATCGCCTCCTGCAGCGGGGGCGGCCTGGAGACGGGCAACAGCGAGGAAGAGGAGGACGCGCCGGCCGTCATCTCCGACTTCATCTACCAGGGCGCCAGCCTGCACAACCAGACAGACCGCACGGGCGAGACCGCCTTGCACCTGGCCGCCCGCTACTCACGCTCTGATGCCGCCAAGCGCCTGCTGGAGGCCAGCGCAGATGCCAACATCCAGGACAACATGGGCCGCACCCCGCTGCATGCGGCTGTGTCTGCCGACGCACAAGGTGTCTTCCAGATCCTGATCCGGAACCGAGCCACAGACCTGGATGCCCGCATGCATGATGGCACGACGCCACTGATCCTGGCTGCCCGCCTGGCCGTGGAGGGCATGCTGGAGGACCTCATCAACTCACACGCCGACGTCAACGCCGTAGATGACCTGGGCAAGTCCGCCCTGCACTGGGCCGCCGCCGTGAACAATGTGGATGCCGCAGTTGTGCTCCTGAAGAACGGGGCTAACAAAGATATGCAGAACAACAGGGAGGAGACACCCCTGTTTCTGGCCGCCCGGGAGGGCAGCTACGAGACCGCCAAGGTGCTGCTGGACCACTTTGCCAACCGGGACATCACGGATCATATGGACCGCCTGCCGCGCGACATCGCACAGGAGCGCATGCATCACGACATCGTGAGGCTGCTGGACGAGTACAACCTGGTGCGCAGCCCGCAGCTGCACGGAGCCCCGCTGGGGGGCACGCCCACCCTGTCGCCCCCGCTCTGCTCGCCCAACGGCTACCTGGGCAGCCTCAAGCCCGGCGTGCAGGGCAAGAAGGTCCGCAAGCCCAGCAGCAAAGGCCTGGCCTGTGGAAGCAAGGAGGCCAAGGACCTCAAGGCACGGAGGAAGAAGTCCCAGGACGGCAAGGGCTGCCTGCTGGACAGCTCCGGCATGCTCTCGCCCGTGGACTCCCTGGAGTCACCCCATGGCTACCTGTCAGACGTGGCCTCGCCGCCACTGCTGCCCTCCCCGTTCCAGCAGTCTCCGTCCGTGCCCCTCAACCACCTGCCTGGGATGCCCGACACCCACCTGGGCATCGGGCACCTGAACGTGGCGGCCAAGCCCGAGATGGCGGCGCTGGGTGGGGGCGGCCGGCTGGCCTTTGAGACTGGCCCACCTCGTCTCTCCCACCTGCCTGTGGCCTCTGGCACCAGCACCGTCCTGGGCTCCAGCAGCGGAGGGGCCCTGAATTTCACTGTGGGCGGGTCCACCAGTTTGAATGGTCAATGCGAGTGGCTGTCCCGGCTGCAGAGCGGCATGGTGCCGAACCAATACAACCCTCTGCGGGGGAGTGTGGCACCAGGCCCCCTGAGCACACAGGCCCCCTCCCTGCAGCATGGCATGGTAGGCCCGCTGCACAGTAGCCTTGCTGCCAGCGCCCTGTCCCAGATGATGAGCTACCAGGGCCTGCCCAGCACCCGGCTGGCCACCCAGCCTCACCTGGTGCAGACCCAGCAGGTGCAGCCACAAAACTTACAGATGCAGCAGCAGAACCTGCAGCCAGCAAACATCCAGCAGCAGCAAAGCCTGCAGCCGCCACCACCACCACCACAGCCGCACCTTGGCGTGAGCTCAGCAGCCAGCGGCCACCTGGGCCGGAGCTTCCTGAGTGGAGAGCCGAGCCAGGCAGACGTGCAGCCACTGGGCCCCAGCAGCCTGGCGGTGCACACTATTCTGCCCCAGGAGAGCCCCGCCCTGCCCACGTCGCTGCCATCCTCGCTGGTCCCACCCGTGACCGCAGCCCAGTTCCTGACGCCCCCCTCGCAGCACAGCTACTCCTCGCCTGTGGACAACACCCCCAGCCACCAGCTACAGGTGCCTGAGCACCCCTTCCTCACCCCGTCCCCTGAGTCCCCTGACCAGTGGTCCAGCTCGTCCCCGCATTCCAACGTCTCCGACTGGTCCGAGGGCGTCTCCAGCCCTCCCACCAGCATGCAGTCCCAGATCGCCCGCATTCCGGAGGCCTTCAAG |
| Nucleotide Sequence of RNF187 | CTGGCGCTCCCTGCGGGCCCCGCCGAGGCCGCCTGCGCCCTGTGCCAGCGCGCGCCCCGGGAACCGGTGCGCGCCGACTGCGGCCACCGCTTCTGTCGGGCGTGCGTGGTGCGCTTCTGGGCCGAGGAGGACGGGCCCTTCCCGTGCCCCGAGTGCGCCGACGACTGCTGGCAGCGCGCCGTGGAGCCCGGCAGGCCCCCGCTCAGCCGCCGCCTTCTGGCGCTCGAGGAGGCGGCCGCGGCGCCCGCGCGCGACGGCCCGGCCAGCGAGGCCGCGCTGCAGCTGCTGTGCCGCGCCGACGCCGGCCCGCTCTGCGCCGCCTGCCGTATGGCTGCGGGCCCCGAGCCGCCCGAGTGGGAACCGCGCTGGAGGAAGGCGCTGCGCGGCAAGGAGAACAAGGGGTCTGTGGAAATCATGAGAAAGGACTTGAATGACGCCCGGGACCTGCATGGCCAGGCAGAGTCAGCAGCTGCAGTGTGGAAGGGACACGTGATGGACCGTAGGAAGAAGGCACTGACCGACTACAAGAAGCTGCGGGCCTTCTTTGTGGAGGAGGAGGAGCATTTCCTGCAGGAGGCTGAGAAGGAGGAGGGGCTCCCTGAGGACGAGCTGGCTGACCCCACTGAGCGGTTCAGGTCACTGCTGCAGGCGGTCTCGGAGCTGGAGAAGAAGCATCGCAACCTGGGCCTCAGCATGCTGCTGCAGTGA |
