# Supplementary figures and images for: An essential role of RNF187 in Notch1 mediated metastasis of hepatocellular carcinoma
Source: J Exp Clin Cancer Res. 2019 Sep 2;38:384. doi: 10.1186/s13046-019-1382-x (PMC6720101; doi:10.1186/s13046-019-1382-x)

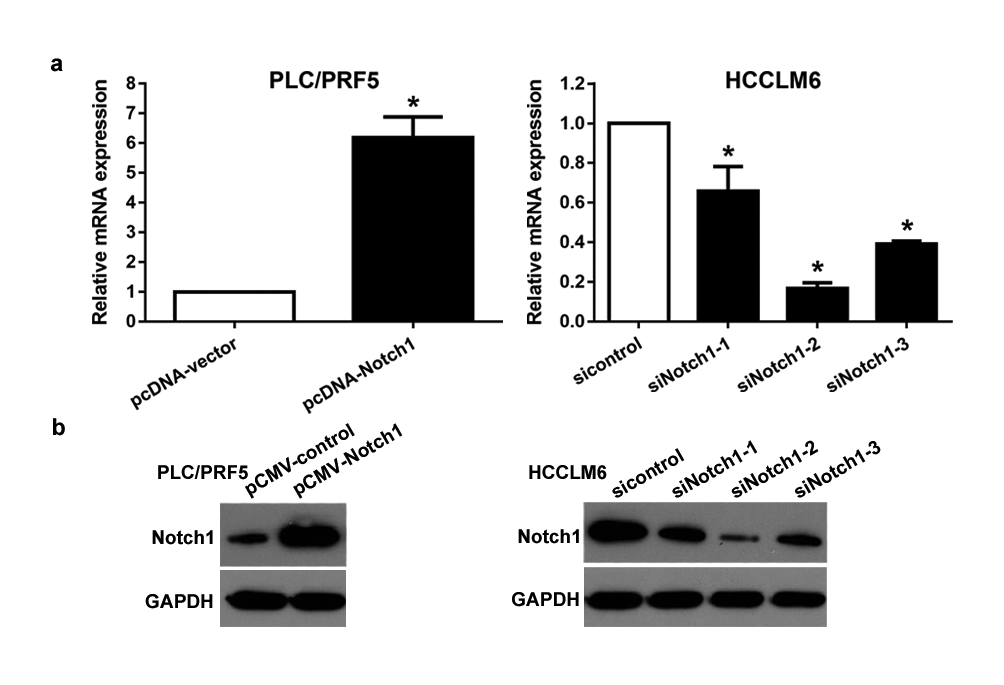

Supplement: Supplementary file 2 — Figure S1. The efficacy of Notch1 silence and ectopic expression is determined in HCC cells. (a) Real-time PCR and (b) Western blotting assays confirmation of Notch1 mRNA and protein expression in PLC/PRF5-pcDNA-Notch1 cells, HCCLM6-siNotch1 cells and their control cells. *: P < 0.05. (TIF 85 kb) [file 13046_2019_1382_MOESM2_ESM.tif]

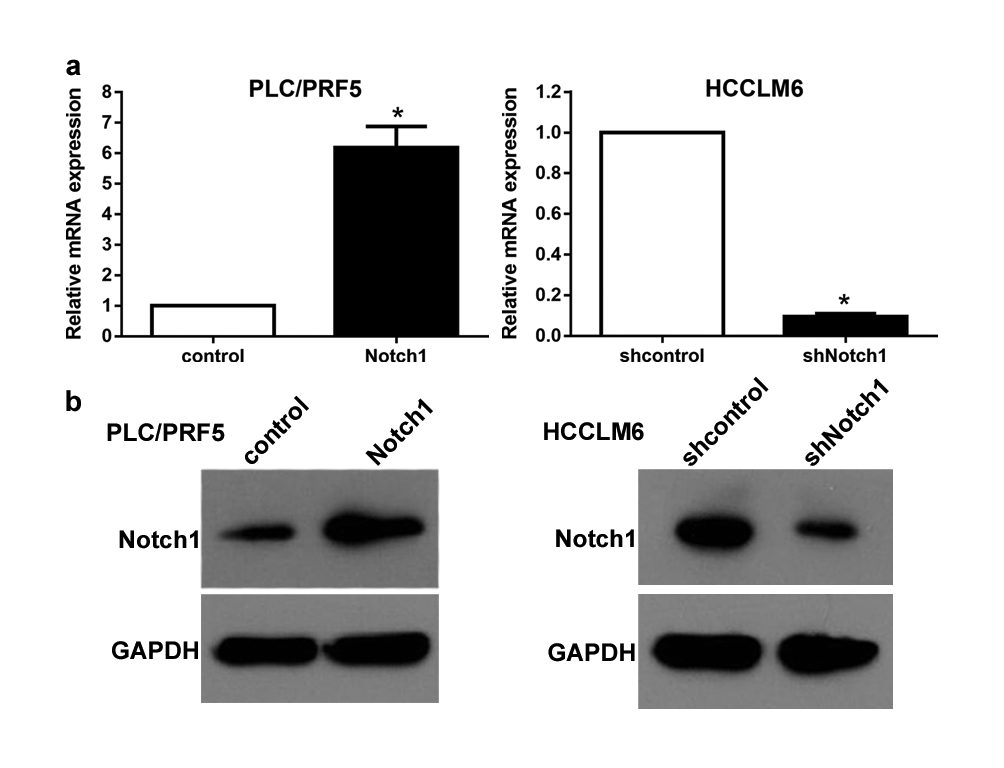

Supplement: Supplementary file 3 — Figure S2. The efficacy of Notch1 silence or ectopic expression is determined in stably transfected HCC cells. (a) Real-time PCR and (b) Western blotting assays confirmation of Notch1 mRNA and protein expression in PLC/PRF5- Notch1 cells, HCCLM6-shNotch1 cells and their control cells. *: P < 0.05. (TIF 98 kb) [file 13046_2019_1382_MOESM3_ESM.tif]

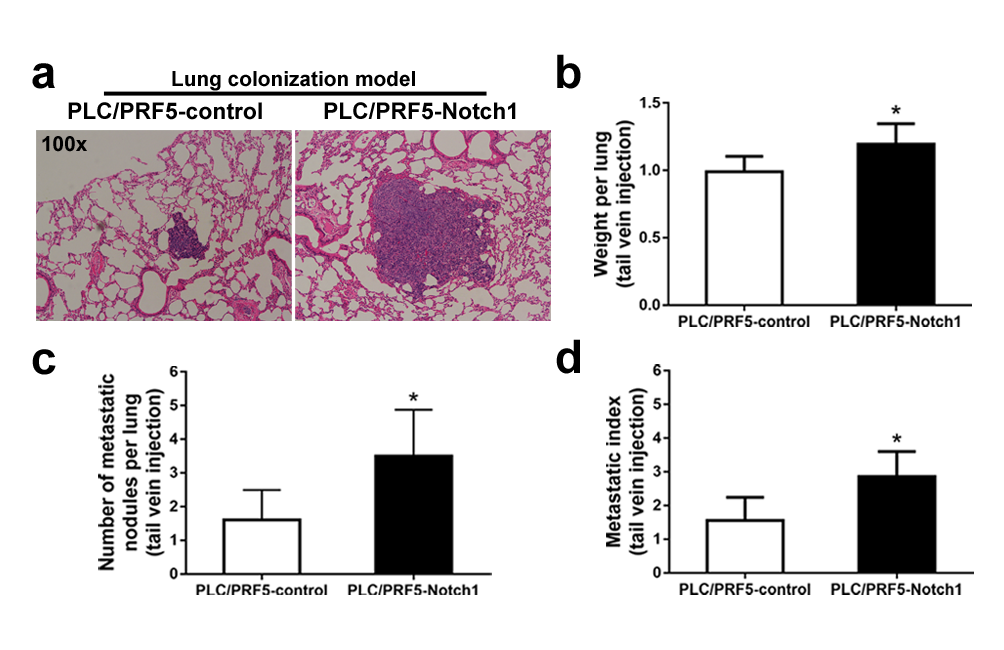

Supplement: Supplementary file 4 — Figure S3. In vivo lung colonization assays. The indicated stable transfectant cells were injected to nude mice via tail vein. (a) H&E staining of the lung metastatic tumors. (b) Lung weights, (c) metastatic nodules and (d) metastasis index of nude mice in each group. *: P < 0.05. (TIF 375 kb) [file 13046_2019_1382_MOESM4_ESM.tif]

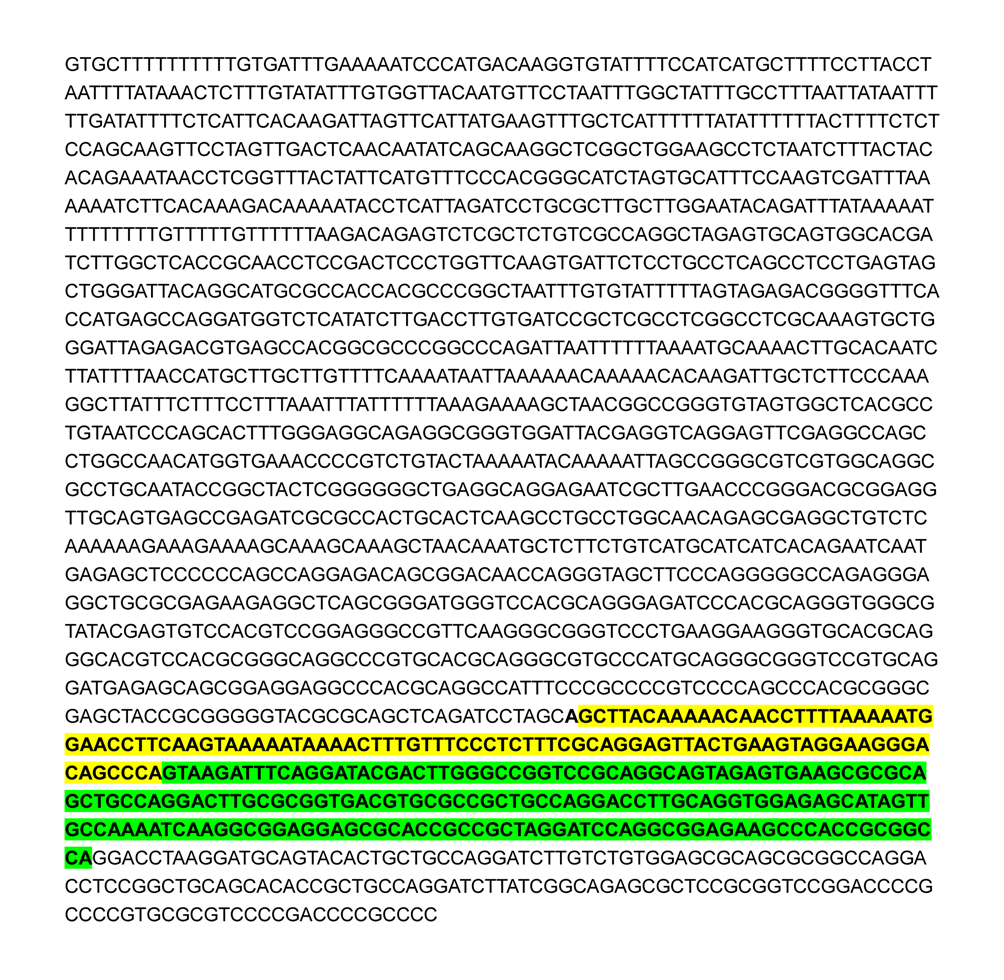

Supplement: Supplementary file 6 — Figure S4. Prediction of Notch1 binding sites at promoter regions of RNF187. Prediction of transcription factor binding sites at promoter regions of RNF187 based on the Chip-seq database of Notch1 binding from GEO (accession no. GSE92701). Yellow highlight text is binding site 1, and green is binding site 2. (TIF 482 kb) [file 13046_2019_1382_MOESM6_ESM.tif]

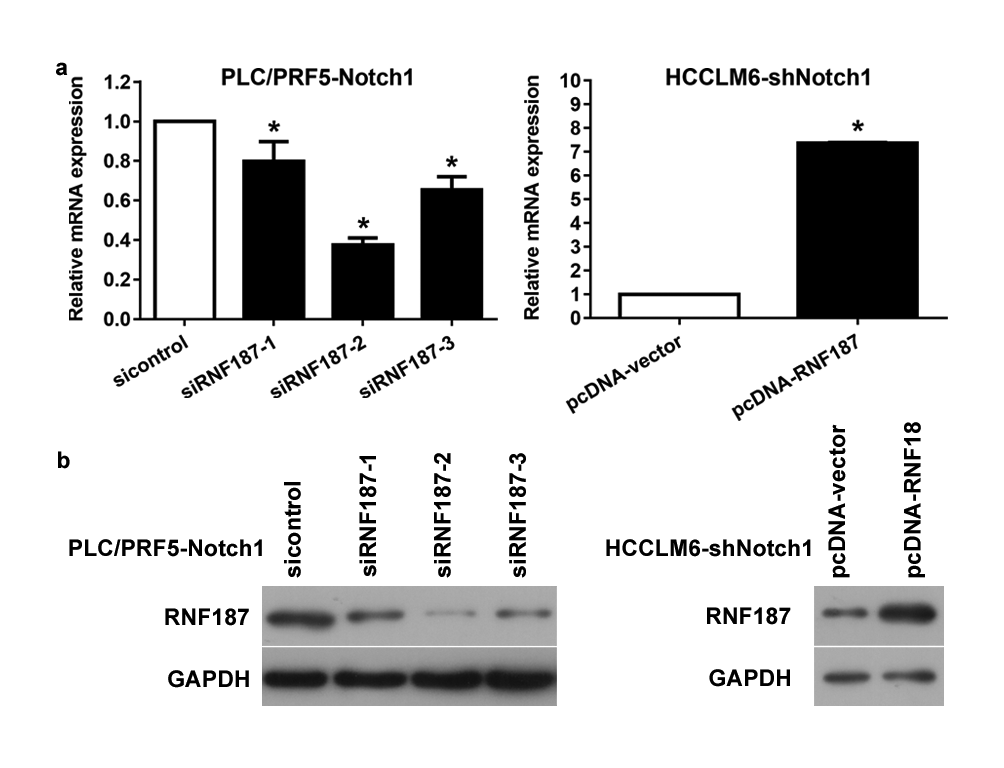

Supplement: Supplementary file 7 — Figure S5. The efficacy of RNF187 silence or ectopic expression is determined in Notch1 mediated HCC cells. (a) Real-time PCR and (b) Western blotting assays confirmation of RNF187 mRNA and protein expression in RNF187 knockdown PLC/PRF5-Notch1 cells, RNF187 ectopic expression HCCLM6-shNotch1 cells and their control cells. *: P < 0.05. (TIF 97 kb) [file 13046_2019_1382_MOESM7_ESM.tif]
